# Robust volume-targeted balanced steady-state free-precession coronary magnetic resonance angiography in a breathhold at 3.0 Tesla: a reproducibility study

**DOI:** 10.1186/1532-429X-16-27

**Published:** 2014-04-23

**Authors:** Sahar Soleimanifard, Matthias Stuber, Allison G Hays, Robert G Weiss, Michael Schär

**Affiliations:** 1 Department of Electrical and Computer Engineering, Johns Hopkins University, Baltimore, MD, USA; 2 Department of Radiology, Centre Hospitalier Universitaire Vaudois, Center for Biomedical Imaging (CIBM) and University of Lausanne, Lausanne, Switzerland; 3 Russell H. Morgan Department of Radiology and Radiological Science, Division of Magnetic Resonance Research, Johns Hopkins University, Baltimore, MD, USA; 4 Department of Medicine, Division of Cardiology, Johns Hopkins University, Baltimore, MD, USA; 5 Philips Healthcare, Cleveland, OH, USA; 6 Barrow Neurological Institute, Keller Center for Imaging Innovation, 350 W. Thomas Rd, Phoenix, AZ 85013, USA

**Keywords:** Coronary artery angiography, 3.0 T magnetic resonance imaging, Balanced steady-state free-precession, Reproducibility, Image-based shimming

## Abstract

**Background:**

Transient balanced steady-state free-precession (bSSFP) has shown substantial promise for noninvasive assessment of coronary arteries but its utilization at 3.0 T and above has been hampered by susceptibility to field inhomogeneities that degrade image quality. The purpose of this work was to refine, implement, and test a robust, practical single-breathhold bSSFP coronary MRA sequence at 3.0 T and to test the reproducibility of the technique.

**Methods:**

A 3D, volume-targeted, high-resolution bSSFP sequence was implemented. Localized image-based shimming was performed to minimize inhomogeneities of both the static magnetic field and the radio frequency excitation field. Fifteen healthy volunteers and three patients with coronary artery disease underwent examination with the bSSFP sequence (scan time = 20.5 ± 2.0 seconds), and acquisitions were repeated in nine subjects. The images were quantitatively analyzed using a semi-automated software tool, and the repeatability and reproducibility of measurements were determined using regression analysis and intra-class correlation coefficient (ICC), in a blinded manner.

**Results:**

The 3D bSSFP sequence provided uniform, high-quality depiction of coronary arteries (n = 20). The average visible vessel length of 100.5 ± 6.3 mm and sharpness of 55 ± 2% compared favorably with earlier reported navigator-gated bSSFP and gradient echo sequences at 3.0 T. Length measurements demonstrated a highly statistically significant degree of inter-observer (r = 0.994, ICC = 0.993), intra-observer (r = 0.894, ICC = 0.896), and inter-scan concordance (r = 0.980, ICC = 0.974). Furthermore, ICC values demonstrated excellent intra-observer, inter-observer, and inter-scan agreement for vessel diameter measurements (ICC = 0.987, 0.976, and 0.961, respectively), and vessel sharpness values (ICC = 0.989, 0.938, and 0.904, respectively).

**Conclusions:**

The 3D bSSFP acquisition, using a state-of-the-art MR scanner equipped with recently available technologies such as multi-transmit, 32-channel cardiac coil, and localized B_0_ and B_1_+ shimming, allows accelerated and reproducible multi-segment assessment of the major coronary arteries at 3.0 T in a single breathhold. This rapid sequence may be especially useful for functional imaging of the coronaries where the acquisition time is limited by the stress duration and in cases where low navigator-gating efficiency prohibits acquisition of a free breathing scan in a reasonable time period.

## Background

Coronary magnetic resonance angiography (CMRA), free of ionizing radiation, has provided a promising means for noninvasive assessment of coronary artery disease (CAD) [1]. Particularly, transient balanced steady-state free-precession (bSSFP) imaging [2] has shown substantial promise towards this goal. This sequence is often considered the method of choice for CMRA at 1.5 T [3-5] due to its high intrinsic blood signal intensity and blood-myocardium contrast requiring no exogenous contrast agent administration [6,7]. The increasing availability of MRI scanners with a static magnetic field (B_0_) strength of 3.0 T and their ability to overcome some of the challenges of 1.5 T scanners have resulted in further efforts to develop 3.0 T CMRA techniques [8-10]. The increased magnetic field strength provides higher signal-to-noise ratio (SNR), which can be exchanged for faster imaging, improved spatial or temporal resolution. These improvements, however, often come with substantial drawbacks. High field strength results in more pronounced B_0_ field inhomogeneities and radio frequency (RF) transmit field (B_1_+) distortions [11,12], both of which degrade image quality and increase tissue energy absorption limiting application of certain sequences. The bSSFP acquisition is especially susceptible to high field artifacts [13,14] and its potential improvements in SNR and contrast-to-noise ratio are hampered by variable image quality at 3.0 T [15]. The bSSFP sequence is also reported to have inferior performance such as shorter arterial visible length, and higher inter-observer variability compared to conventional gradient echo techniques at 3.0 T, in contradiction to their relative performance at 1.5 T [16]. Modified bSSFP sequences have been recently developed for CMRA such as wideband bSSFP [17], which allow for high field off-resonance artifact suppression. However these modified implementations also come with notable drawbacks such as longer scan time and lower SNR compared with the conventional bSSFP [17]. Alternate acquisition techniques such as radial bSSFP [18] have been reported to achieve an improved image quality and vessel sharpness compared to Cartesian bSSFP. However, adoption of these navigator-gated techniques with 7–12 minutes of scan time [18] is still quite limited at 3.0 T. In fact, the challenging implementation of high field bSSFP has led to utilization of spoiled gradient echo techniques at 3.0 T, sometimes requiring the use of exogenous contrast agents [19].

Recent advances in hardware and software may nonetheless fill this gap and improve the previously mixed performance of bSSFP at 3.0 T. B_0_ inhomogeneities linearly increase with field strength but can be sufficiently attenuated by localized second-order shimming and on-resonant frequency f_0_ determination [20]. Parallel excitation with multi-channel RF transmit systems are reported to better manage RF power deposition and provide a more homogeneous B_1_+ field, facilitating the desired RF excitation angles in the heart [21,22]. A recent quantitative evaluation of B_1_+ map before and after local RF shimming demonstrates that signal variations in cardiac bSSFP at 3.0 T are subject-specific. The study concludes that local RF shimming can significantly reduce such variations and improve the image quality of bSSFP at 3.0 T [23]. Additionally, the 32-channel phased-array coil provides an SNR increase of as much as 40% over conventional cardiac-optimized phased array coils, enhances image quality, and improves delineation of the coronary arteries [24]. Therefore, in this study, we aimed to utilize recently available advanced hardware and software to minimize the susceptibility off-resonance artifacts and improve the image quality at 3.0 T, and implement a robust and accelerated single-breathhold bSSFP coronary methodology. Subsequently, we sought to evaluate the performance of the developed sequence as well as its reproducibility in humans. To the best of our knowledge, the inter-scan reproducibility of bSSFP sequence for CMRA has not been previously investigated at 3.0 T. This rapid sequence should be important for functional imaging of the coronaries where the acquisition time is limited by the stress duration [25-27] and in cases where low navigator-gating efficiency prohibits acquisition of a free breathing scan in a reasonable time period [3].

## Methods

### Study population

Fifteen healthy adults with no history of cardiovascular disease and three patients with stable coronary artery disease (CAD), documented with at least one 50% lesion on clinically indicated cardiac catheterization within the prior six months, were enrolled in the study. All eighteen subjects underwent one CMRA examination. Additionally, eight volunteers and one patient underwent a second CMRA examination to study reproducibility of the bSSFP sequence. The subjects in the reproducibility sub-group were removed from the scanner after the first examination. They were returned to, and repositioned in, the scanner after a 15-minute rest period and the complete examination was repeated. The protocol was approved by the Johns Hopkins Institutional Review Board and written informed consent was obtained from all participants.

### CMRA protocol and transient bSSFP sequence

All the studies were performed on a commercial whole body 3.0 T MR scanner (Achieva R3.2, Philips Healthcare, Best, The Netherlands) equipped with multi-transmit system, vector electrocardiography triggering [28], and a 32-channel cardiac phased-array coil. The imaging protocol began with a multi-slice segmented *k-space* gradient echo scout scan in transverse, sagittal, and coronal views to identify the heart and the lung-liver interface for navigator localization. Next, a B_1_+ calibration scan [29] was acquired for localized B_1_+ shimming and a more homogeneous transmit field in the heart in the subsequent scans. The calibration scan was followed by a sensitivity encoding (SENSE) reference scan [30]. Subsequently, an axial mid-ventricular bSSFP cine scan was obtained during free breathing to visually identify the period of minimal coronary motion. The beginning of this stationary period was chosen as trigger delay for subsequent scans in the protocol. The cine scan was followed by a quick low spatial resolution free breathing navigator-gated and corrected whole-heart three-dimensional (3D) coronary localizer scan in the transverse plane. The three-point plan tool [31] was utilized on this scout scan for planning the subsequent 3D acquisitions. Next, a fast B_0_-map acquisition was performed allowing determination of localized second-order shim corrections and on-resonance f_0_ frequency to improve B_0_ field homogeneity and to reduce potential bSSFP off-resonance artifacts in the coronary arterial tree [20]. Three-dimensional CMRA acquisitions were then performed along the 3D track of the coronaries of interest using the volume-targeted bSSFP sequence with centric *k-space* profile ordering, and a half-Fourier-acquisition factor of 0.6. A half-alpha TR-half preparation pulse followed by 10 startup RF pulses were used to accelerate the approach to steady state [32]. A SENSE acceleration factor of 2.5 with an additional oversampling factor of 1.3 was used in the phase encoding direction. A spectrally selective saturation pulse preceded the data acquisition window for fat suppression. Scan parameters were the following: repetition/echo times were 3.9/1.9 ms, RF excitation angle 50°, standard Sinc-Gaussian RF pulse with time-bandwidth product of 6, field-of-view 300 × 300 × 20 mm^3^, acquired voxel size 1.0 × 1.0 × 2.0 mm^3^, reconstructed voxel size 0.8 × 0.8 × 1.0 mm^3^, acquisition window 105 ms. The data were acquired during one breathhold for respiratory motion suppression. The duration of each acquired scan was recorded, and the total examination time was measured accordingly.

### Image analysis

The analysis of bSSFP scans was performed along the entire visualized course of each artery using Soap-Bubble, a previously reported interactive coronary visualization and analysis tool [33]. Using visually identified points throughout the 3D track of coronaries and multi-planar reformatting of the 3D CMRA images, vessel length as well as average vessel diameter and sharpness within the visualized course of each artery were measured on each dataset, as previously described [33].

To assess intra-observer variability, the first CMRA examinations in eighteen subjects were analyzed twice by observer 1 (SS) in a blinded manner. Additionally, blinded and independent analyses were performed in these scans by observer 2 (MS) to evaluate inter-observer variability. Furthermore, scans obtained during the second successive examination in nine volunteers were analyzed by observer 1 to measure inter-scan variability (the scans in the first examination were considered the reference standard). Care was taken to ensure that the vessel diameter and sharpness measurements were performed at the same anatomical levels, and for example, observer 1 reported to observer 2 the distance from the ostium where the semi-automated measurements began.

### Statistical analysis

All datasets were included in the analysis, and results reported as mean ± one standard error of mean. Intra-observer (observer 1 repeat measurements, n = 20) and inter-observer (observer 1 vs. observer 2 measurements, n = 20), and inter-scan (first scan vs. repeat scan observer 1 measurements, n = 9) agreements were assessed using Pearson’s correlation coefficient, Bland-Altman analysis and intra-class correlation coefficient (ICC) [34], the proportion of total variability accounted for by the variability among observers. If the coefficient is high, it means only a small portion of the variability is due to variability in measurement on different occasions; hence, the reproducibility is high. A *p*-value < 0.05 was considered statistically significant in all analyses.

## Results

**Figure 1:**
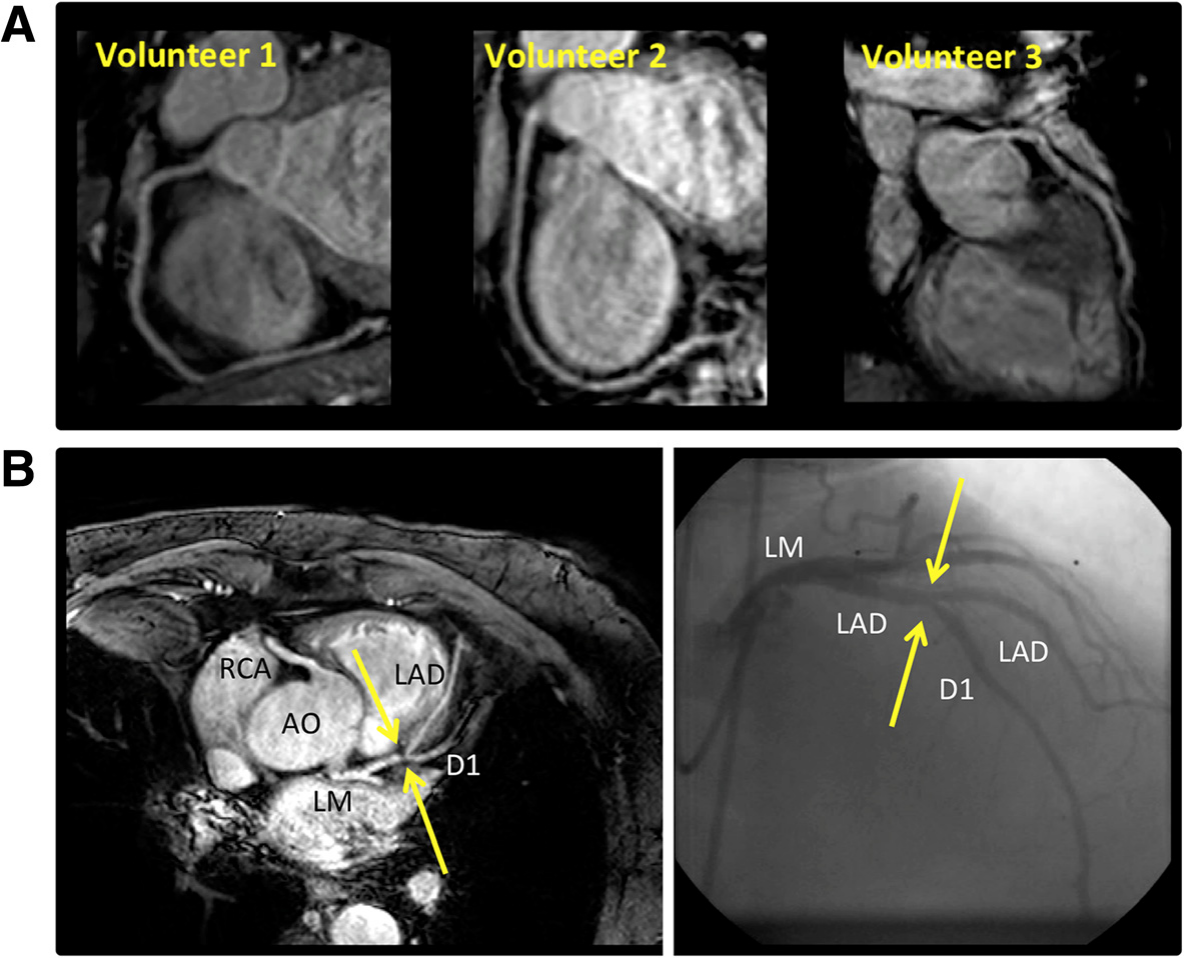
**Double oblique single-breathhold 3D bSSFP coronary magnetic resonance angiograms of the right coronary artery and left anterior descending in three healthy volunteers (A) and in a 66-year-old patient (B) with 50-70% lesion at the diagonal branch of the LAD identified using diagnostic x-ray angiogram.** AO: Aorta, RCA: Right Coronary Artery. LM: Left Main Artery. LAD: Left Anterior Descending Artery. D1: First Diagonal Branch. MRA Images were multi-planar reformatted.

**Figure 2:**
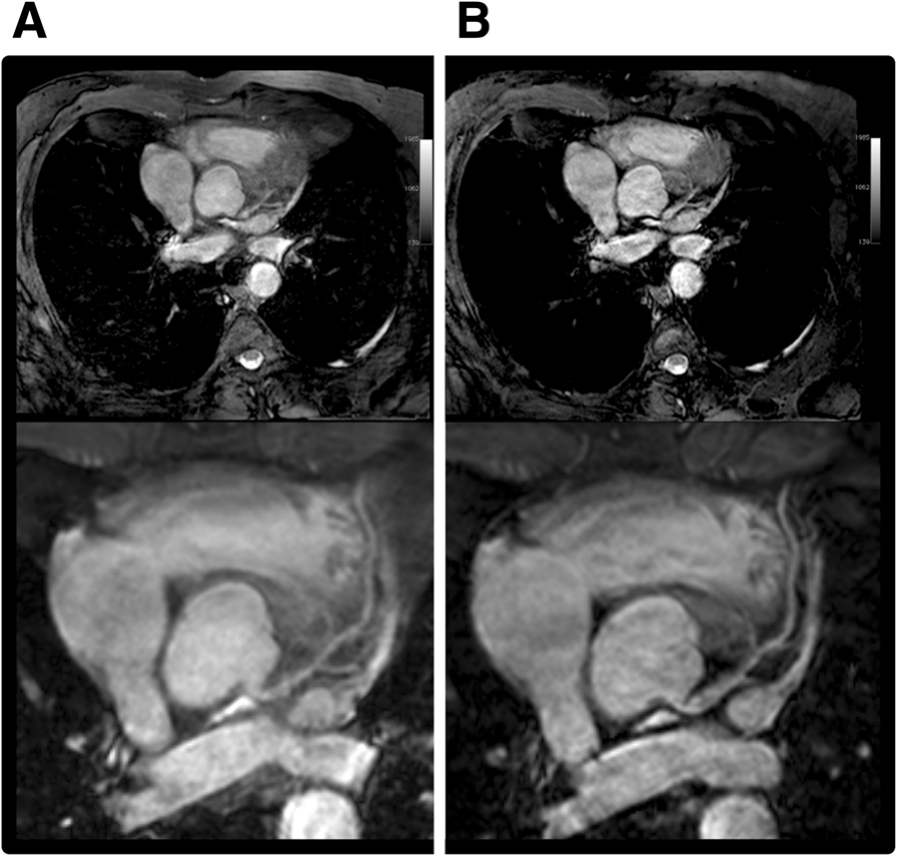
**Volume-targeted bSSFP scans of the left anterior descending artery in a healthy volunteer, acquired during free breathing (A) and during one breathhold (B), with the same spatial coverage and resolution.** Top: 2D slice from the 3D volume selected for demonstration. Bottom: multi-planar reformatted images of the artery.

All 18 subjects (age 21–75 years (mean ± SD: 38 ± 18); 8 women) including the 3 CAD patients (age 65–75 years (mean ± SD: 71 ± 5); 1 woman) successfully completed the CMRA examination. All subjects were in stable sinus rhythm and the average heart rate was 70 ± 12 beats per minute. A total of 20 coronary arteries (right coronary artery (RCA): n = 15; left anterior descending (LAD): n = 5) were imaged. Additionally, 9 subjects completed a repeated examination (8 healthy, age 21–51 years (mean ± SD: 30 ± 9); 1 patient, age 72 years) and their 9 coronary arteries (RCA: n = 7; LAD: n = 2) were successfully imaged twice. The total duration of each examination including the acquisition of scouts, reference, and calibration scans as well as geometry planning was 13 ± 3 minutes. The average duration of the low resolution scout MRA scan, acquired with respiratory navigator-gating, was 76 ± 15 sec (n = 18). The total duration of B_1_ and B_0_ calibration scans as well as the time required for local determination of on-resonant f_0_ were 1–2 minutes. Localization of each coronary artery of interest took 2–3 minutes, and the duration of each bSSFP sequence was 20.5 ± 2.0 seconds. Figure 1 shows examples of multi-planar reformatted [33] images of RCA and LAD in (A) three volunteers and (B) one patient with a 50-70% lesion at the diagonal branch of the LAD, as identified on a diagnostic x-ray angiogram. Figure 2 illustrates an example of the proposed breathhold acquisition and its navigator-gated and -corrected counterpart with the same spatial resolution and coverage in a 52-year-old healthy man. Using SENSE factor of 1.5 in the phase-encode direction and no half-Fourier acquisition, the free-breathing scan was completed in 3 minutes and 44 seconds (average navigator efficiency = 24%, gating window = 5 mm).

The single breathhold approach achieved an average visualized vessel length of 100.5 ± 6.3 mm (n = 20), which was used for quantitative analysis of vessel sharpness and diameters as shown in Table 1. The mean vessel diameter was 2.8 ± 0.1 mm averaged over the visualized length of coronary arteries and 3.1 ± 0.1 mm for the proximal 4 cm segment. Mean vessel sharpness was 55 ± 2% over the visualized coronary length and 56 ± 2% in the proximal 4 cm segment (Table 1).

**Table 1:** Vessel length, diameter, and vessel sharpness averaged in 18 subjects (20 coronary arteries: RCA = 15, LAD = 5)

	**Intra-observer (n = 20)**			**Inter-observer (n = 20)**		**Inter-scan (n = 9)**		
	**Reference analysis**	**Repeat analysis**	**ICC**	**Analysis**	**ICC**	**Reference analysis (subset)**	**Repeat scan analysis**	**ICC**
Length [mm]	100.5 ± 6.3	101.6 ± 6.1	0.993	98.7 ± 6.6	0.896	95.7 ± 10.0	98.2 ± 9.5	0.974
Diameter [mm]								
Proximal 40 mm	3.1 ± 0.1	3.1 ± 0.1	0.986	3.1 ± 0.1	0.979	3.0 ± 0.1	3.0 ± 0.1	0.952
Full length	2.8 ± 0.1	2.8 ± 0.1	0.987	2.8 ± 0.1	0.976	2.7 ± 0.1	2.8 ± 0.1	0.961
Sharpness [%]								
Proximal 40 mm	56 ± 2	55 ± 2	0.979	56 ± 2	0.972	57 ± 2	55 ± 3	0.736
Full length	55 ± 2	54 ± 2	0.989	56 ± 1	0.938	55 ± 3	54 ± 3	0.905

Data are presented as mean ± standard error of mean. ICC = intra-class correlation coefficient.

**Figure 3:**
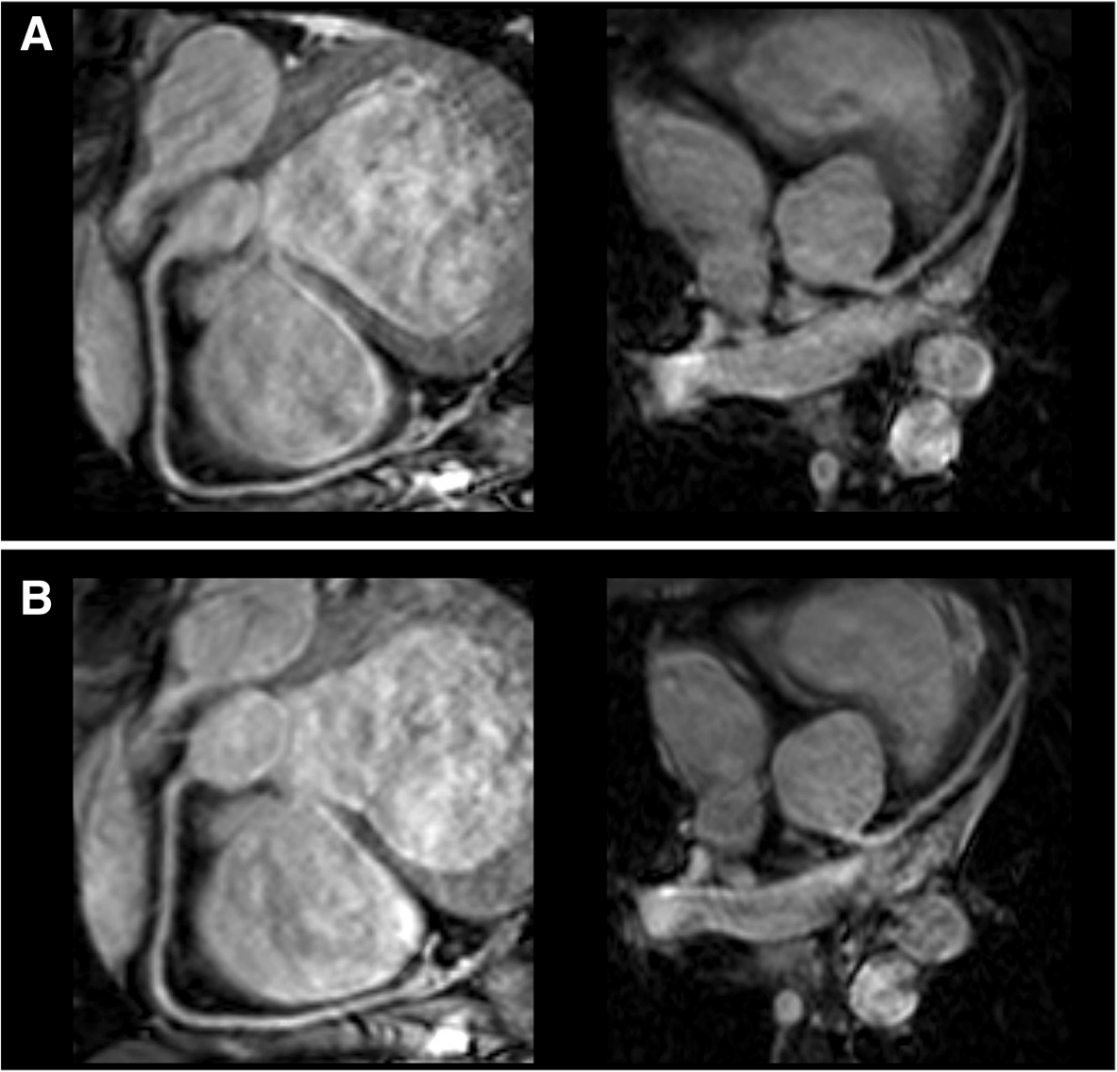
**Double oblique single-breathhold 3D bSSFP coronary magnetic resonance angiograms of the right coronary artery and left anterior descending in two healthy volunteers obtained during two separate scanning sessions: reference (A), repeat (B).** Images were multi-planar reformatted.

**Figure 4:**
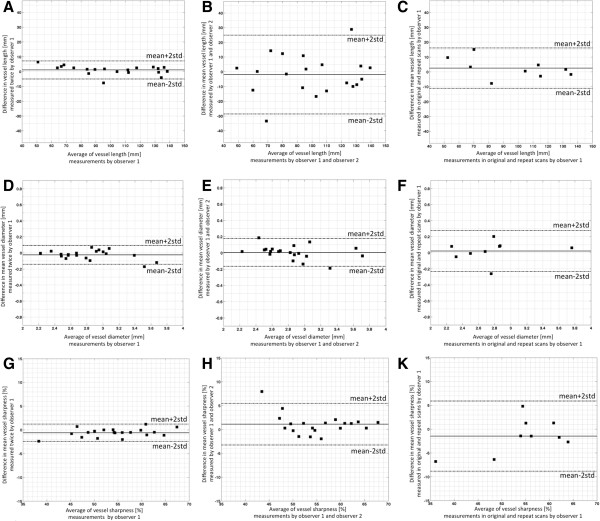
**Repeatability and reproducibility of vessel length, mean diameter, mean sharpness measurements with bSSFP sequence.** Bland-Altman plot shows good **(A)** intra-observer (r = 0.994), **(B)** inter-observer (r = 0.894), and **(C)** intra-class (r = 0.980) agreement between length measurements [mm] with low variability. Bland-Altman plot shows good **(D)** intra-observer (r = 0.990), **(E)** inter-observer (r = 0.977), and **(F)** intra-class (r = 0.959) agreement between mean diameter measurements [mm] with low variability. Bland-Altman plot shows good **(G)** intra-observer (r = 0.993), **(H)** inter-observer (r = 0.953), and **(K)** intra-class (r = 0.932) agreement between mean sharpness measurements [%] with low variability.

Figure 3 shows examples of multi-planar reformatted images of RCA and LAD obtained during separate scanning sessions in two healthy volunteers. The mean vessel length (original and repeat values) for the subjects who underwent the scans twice measured 95.7 ± 10.5 mm and 98.2 ± 9.5 mm, respectively (p = 0.298). In this subset of volunteers, the average diameter of coronary arteries was measured 2.7 ± 0.1 mm and 2.8 ± 0.1 mm in the original and repeat examinations, respectively (p = 0.609). Average vessel sharpness values from the two scans were also found in strong concordance (original: 55 ± 3%, repeat: 54 ± 3%; p = 0.269) between the reference and repeated analyses of observer 1 as well as the reference analysis of observer 1 and the analysis of observer 2 were excellent for vessel length, diameter and vessel sharpness measurements (Table 1). Using Pearson’s correlation coefficient, there was a highly statistically significant intra-observer (r = 0.994, R^2^ = 98.9%, SE = 0.28), inter-observer (r = 0.894, R^2^ = 80.0%, SE = 1.36) and inter-scan (r = 0.980, R^2^ = 96.1%, SE = 0.60) agreement for vessel length measurements. The results of the Bland–Altman analysis demonstrating intra-observer, inter-observer, and inter- scan agreements are shown in Figure 4A, B and C. Additionally, ICC values demonstrated a high degree of intra-observer, inter-observer, and inter-scan concordance for length measurements (ICC = 0.993, 0.896, and 0.974, respectively). Similarly, a large degree of agreement was observed for mean vessel diameter measurements (intra-observer: r = 0.990, R^2^ = 98.0%, SE = 0.05, ICC = 0.987; inter-observer: r = 0.977, R^2^ = 95.5%, SE = 0.08, ICC = 0.976; intra-scan: r = 0.959, R^2^ = 92.0%, SE = 0.14, ICC = 0.961). Figure 4D,E and F illustrate the Bland-Altman analysis results for diameter measures. Lastly, vessel sharpness values revealed high degree of reproducibility as well (intra-observer: r = 0.993, R^2^ = 98.7%, SE = 0.88, ICC = 0.989; inter-observer: r = 0.953, R^2^ = 90.8%, SE = 2.00, ICC = 0.938; intra-scan: r = 0.932, R^2^ = 86.9%, SE = 3.68, ICC = 0.905). Figure 4G, H and K summarize the results of Bland-Altman analysis for vessel sharpness measurements.

## Discussion

Fat saturated, segmented bSSFP imaging has been widely used in cardiac MR imaging because it produces images with inherently higher signal and contrast than conventional gradient echo counterparts, and it does not require the administration of contrast agents to yield improved SNR and spatial resolution. bSSFP at 1.5 T has been evaluated in healthy and patient populations with X-ray angiographic correlation, and reported to noninvasively detect CAD with high sensitivity [4,5]. Higher field MR systems offer a theoretically higher SNR, which can be traded for higher spatial resolution or reduced imaging time and thereby reduced motion artifacts, all of which are especially important in coronary imaging. However, high field cardiac imaging entails some major restrictions such as increased RF power deposition and susceptibility-related field inhomogeneities. Because of these limitations, conventional bSSFP imaging in particular has inferior quality and higher variability at higher fields [15,16], which has led to readoption of conventional gradient echo sequences, often requiring administration of contrast agents for a sufficient blood-myocardium contrast [9,19,35].

This study demonstrates that advances in MR hardware and software can address the shortcomings of 3.0 T bSSFP imaging and provide a step forward to a more robust, reproducible, and fast technique with a high intrinsic contrast for 3D imaging of human coronary arteries at higher fields. The MR scanner, used in this study, equipped with a 32-channel phased-array coil and multi-transmit system allowed volume targeted acquisition of 3D images in a single breathhold. 32-channel phased-array coils are reported to have significantly improved SNR and geometry factor, which facilitate use of large parallel imaging acceleration factors [36]. Second-order shimming, reported to be considerably more effective than linear shimming [20], was applied for suppression of B_0_ field inhomogeneities, and on-resonant frequency was determined at the level of the coronary arteries. Multi-transmit technology and localized RF shimming were integrated with higher order shimming and utilized to obtain proper knowledge of the B_1_+−field and accurate estimation of SAR [22]. Although we did not perform a quantitative analysis of the B_1_+−field, subject-specific B_1_ shimming has been shown to significantly enhance the homogeneity of the local field and to achieve more accurate excitation angles [23]. These refinements have been reported to improve the quality of cardiac bSSFP imaging with respect to image homogeneity, diagnostic confidence, and off-resonance artifacts [22]. Combined with parallel imaging and half-Fourier-acquisition imaging of a 300 × 300 × 20 mm^3^ volume with acquired voxel size of 1.0 × 1.0 × 2.0 mm^3^ was supported. This spatial coverage and resolution compared equally or favorably with the prior volume-targeted implementations of bSSFP [15-17] and the common gradient echo sequences at 3.0 T [10,16], as well as with the well-tested volume-targeted implementations of bSSFP at 1.5 T [4,37,38]. The single-breathhold scan with an average duration of 20.5 seconds, was comfortably tolerated by all subjects and achieved with an acquisition window of 105 ms. This temporal resolution too compared favorably with the previously reported single-breathhold implementations of bSSFP with similar spatial resolution and coverage at 1.5 T as well as 3.0 T (acquisition window between 108–150 ms [4,15,17]).

The present implementation of the bSSFP sequence provided high-quality images of arterial lumen with enhanced contrast. The average vessel sharpness of 55 ± 2% compared favorably with bSSFP imaging at 1.5 T as well as gradient echo imaging at 3.0 T (average reported sharpness ranging between 40-46% [37,39]). Reproducible assessment of arterial lumen was provided within a long continuous segment of the vessels with an average visualized vessel length of 100.5 ± 6.3 mm, which too compared favorably with standard bSSFP sequences at 1.5 and 3.0 T (average visible length ranging between 50.0-95.0 mm [4,16]). The mean vessel diameter imaged by this technique was consistent with what has been published in literature for similar cohorts.

Although, the bSSFP sequence has been shown to be a highly reproducible technique for quantitative assessment of major coronary arteries at 1.5 T [37], to the best of our knowledge the reproducibility of this technique at higher fields has never been fully investigated. This study demonstrated that the present 3D bSSFP protocol has a very high degree of concordance in repeat measures of vessel length, diameter, and sharpness (Table 1) with no significant difference between the two observers or the two scans. A comparison of variability in diameter measures reveals that the intra-observer, inter-observer, and inter-scan ICC values from the current study (0.987, 0.976, and 0.961, respectively) are higher than the reported values from 3D bSSFP at 1.5 T (intra-observer, inter-observer, and inter-scan ICC values ranging between 0.89-0.98, 0.89-0.98, and 0.63-0.86, respectively [37]).

These results suggest that single-breathhold 3D bSSFP, successfully tested in healthy volunteers and CAD patients within a diverse age group (ranging between 21–75 years), provides a highly reproducible technique at 3.0 T, with a high spatial resolution and a well-defined time requirement. This rapid approach may facilitate functional 3D CMRA studies during stress conditions [25], which are currently conducted with 2D techniques. Furthermore, it can provide an appealing alternate in cases where the navigator-gated free-breathing counterparts, relying on breathing patterns and diaphragm position, result in prolonged and sometimes unpredictable scanning time. In the worst case, the low navigator efficiency may lead to unsuccessful imaging sessions [3]. In this study, the bSSFP sequence was tested in a targeted volume, which requires prescription of a separate 3D volume along each major coronary artery by a skilled operator. This usually adds about 2–3 minutes of planning per coronary artery to the imaging session. In comparison, whole-heart CMRA considerably improves both volumetric coverage and procedural ease-of-use and can be completed in 7–12 minutes of free-breathing scan time [18]. Nevertheless, volumetric-targeted CMRA continues to be a valuable alternate at higher fields [40]. Yet, it would be important in future work to determine whether the present single-breathhold bSSFP sequence could be adapted to a whole-heart approach. With higher volumetric coverage, scanning time naturally increases if the spatial and temporal resolution remain constant, but it offers the opportunity to exploit 2D rather than 1D SENSE [41]. The boundaries of this strategy remain to be explored in this context but provide a promising opportunity to extend this technique for enhanced volumetric coverage. Clinical evaluation of this current technique and assessment of its diagnostic accuracy also remain to be systematically investigated in a larger cohort of patients with coronary artery disease.

### Limitations

One limitation to this study is that the imaging sequence was not evaluated in both the right and left coronary systems in every volunteer. The coronary artery that was best visualized on the whole-heart scout scan was selected for imaging and when visual image quality was equivalent for the RCA and the LAD, both arteries were selected for imaging.

## Conclusions

This study demonstrates that a 3D bSSFP acquisition, using advances in 3.0 T MR such as multi-transmit system, 32-channel cardiac coil, and localized B_0_ and B_1_+ shimming, provides high-quality noninvasive imaging of the proximal to distal segments of the major coronary arteries in a single breathhold. This accelerated sequence enables highly reproducible assessment of coronary arterial tree in healthy subjects and patients with CAD.

## Competing interests

Michael Schär is a full-time employee of Philips Healthcare, the manufacturer of equipment used in this study.

## Authors’ contributions

SS: made substantial contributions to design of study, acquisition, analysis and interpretation of data, and drafted the manuscript. MS: made substantial contributions to design of study, acquisition and interpretation of data, and revision of manuscript. AGH: made substantial contributions to acquisition of data. RGW: made substantial contributions to design of study, acquisition and interpretation of data, and revision of manuscript. MS: substantial contributions to design of study, acquisition, analysis and interpretation of data, and revision of manuscript. All authors read and approved the final manuscript.

## References

[B1] Kim WY Danias PG Stuber M Flamm SD Plein S Nagel E Langerak SE Weber OM Pedersen EM Schmidt M Botnar RM Manning WJ Coronary Magnetic Resonance Angiography for the Detection of Coronary Stenoses New Engl J Med 2001 345 1863 9 10.1056/NEJMoa010866 11756576

[B2] Oppelt A Graumann R Barfuss H Fischer H Hartl W Schajor W FISP—a new fast MRI sequence Electromedica 1986 54 15 8

[B3] Sakuma H Ichikawa Y Chino S Hirano T Makino K Takeda K Detection of Coronary Artery Stenosis With Whole-Heart Coronary Magnetic Resonance Angiography J Am Coll Cardiol 2006 48 1946 50 10.1016/j.jacc.2006.07.055 17112982

[B4] McCarthy RM Deshpande VS Beohar N Meyers SN Shea SM Green JD Liu X Bi X Pereles FS Finn JP Kobayashi Y Sakuma H Three-Dimensional Breathhold Magnetization-Prepared TrueFISP: A Pilot Study for Magnetic Resonance Imaging of the Coronary Artery Disease Invest Radiol 2007 42 665 70 10.1097/RLI.0b013e3180661a77 17984762 PMC4124003

[B5] Kato S Kitagawa K Ishida N Ishida M Nagata M Ichikawa Y Katahira K Matsumoto Y Seo K Ochiai R Kobayashi Y Sakuma H Assessment of Coronary Artery Disease Using Magnetic Resonance Coronary Angiography: A National Multicenter Trial J Am Coll Cardiol 2010 56 983 91 10.1016/j.jacc.2010.01.071 20828652

[B6] Spuentrup E Bornert P Botnar RM Groen JP Manning WJ Stuber M Navigator-Gated Free-Breathing Three-Dimensional Balanced Fast Field Echo (TrueFISP) Coronary Magnetic Resonance Angiography Invest Radiol 2002 37 637 47 10.1097/00004424-200211000-00008 12393977

[B7] Deshpande VS Shea SM Laub G Simonetti OP Finn JP Li D 3D magnetization-prepared true-FISP: A new technique for imaging coronary arteries Magn Reson Med 2001 46 494 502 10.1002/mrm.1219 11550241

[B8] Stuber M Botnar RM Fischer SE Lamerichs R Smink J Harvey P Manning WJ Preliminary report on in vivo coronary MRA at 3 Tesla in humans Magn Reson Med 2002 48 425 9 10.1002/mrm.10240 12210906

[B9] Liu X Bi X Huang J Jerecic R Carr J Li D Contrast-enhanced whole-heart coronary magnetic resonance angiography at 3.0 t: comparison with steady-state free precession technique at 1.5 t Invest Radiol 2008 43 663 8 10.1097/RLI.0b013e31817ed1ff 18708861

[B10] Sommer T Hackenbroch M Hofer U Schmiedel A Willinek WA Flacke S Gieseke J Träber F Fimmers R Litt H Schild H Coronary MR angiography at 3.0 T versus that at 1.5 t: initial results in patients suspected of having coronary artery disease1 Radiology 2005 234 718 25 10.1148/radiol.2343031784 15665221

[B11] Ibrahim TS Lee R Abduljalil AM Baertlein BA Robitaille P-ML Dielectric resonances and B1 field inhomogeneity in UHFMRI: computational analysis and experimental findings Magn Reson Imaging 2001 19 219 26 10.1016/S0730-725X(01)00300-9 11358660

[B12] Noeske R Seifert F Rhein K-H Rinneberg H Human cardiac imaging at 3 T using phased array coils Magn Reson Med 2000 44 978 82 10.1002/1522-2594(200012)44:6<978::AID-MRM22>3.0.CO;2-9 11108638

[B13] Zur Y Stokar S Bendel P An analysis of fast imaging sequences with steady-state transverse magnetization refocusing Magn Reson Med 1988 6 175 93 10.1002/mrm.1910060206 3367775

[B14] Fuchs F Laub G Othomo K TrueFISP—technical considerations and cardiovascular applications Eur J Radiol 2003 46 28 32 10.1016/S0720-048X(02)00330-3 12648799

[B15] Bi X Deshpande V Simonetti O Laub G Li D Three-dimensional breathhold SSFP coronary MRA: A comparison between 1.5 T and 3.0 T J Magn Reson Imaging 2005 22 206 12 10.1002/jmri.20374 16028242

[B16] Kaul MG Stork A Bansmann PM Nolte-Ernsting C Lund GK Weber C Adam G Evaluation of Balanced Steady-State Free Precession (TrueFISP) and K-space segmented gradient echo sequences for 3D coronary MR angiography with navigator gating at 3 Tesla Fortschr Röntgenstr 2004 176 1560 5 10.1055/s-2004-813629 15497073

[B17] Lee H-L Shankaranarayanan A Pohost GM Nayak KS Improved coronary MR angiography using wideband steady state free precession at 3 tesla with sub-millimeter resolution J Magn Reson Imaging 2010 31 1224 9 10.1002/jmri.22150 20432360 PMC2908593

[B18] Xie J Lai P Bhat H Li D Whole‐heart coronary magnetic resonance angiography at 3.0 T using short‐TR steady‐state free precession, vastly undersampled isotropic projection reconstruction J Magn Reson Imaging 2010 31 1230 5 10.1002/jmri.22140 20432361 PMC2915571

[B19] Yang Q Li K Liu X Bi X Liu Z An J Zhang A Jerecic R Li D Contrast-enhanced whole-heart coronary magnetic resonance angiography at 3.0-T: a comparative study with X-Ray angiography in a single center J Am Coll Cardiol 2009 54 69 76 10.1016/j.jacc.2009.03.016 19555843 PMC2758632

[B20] Schär M Kozerke S Fischer SE Boesiger P Cardiac SSFP imaging at 3 Tesla Magn Reson Med 2004 51 799 806 10.1002/mrm.20024 15065254

[B21] Zhu Y Parallel excitation with an array of transmit coils Magn Reson Med 2004 51 775 84 10.1002/mrm.20011 15065251

[B22] Mueller A Kouwenhoven M Naehle CP Gieseke J Strach K Willinek WA Schild HH Thomas D Dual-source radiofrequency transmission with patient-adaptive local radiofrequency shimming for 3.0-T cardiac MR imaging: initial experience Radiology 2012 263 77 85 10.1148/radiol.11110347 22371610

[B23] Krishnamurthy R Pednekar A Kouwenhoven M Cheong B Muthupillai R Evaluation of a Subject specific dual-transmit approach for improving B1 field homogeneity in cardiovascular magnetic resonance at 3 T J Cardiovasc Magn Reson 2013 15 68 10.1186/1532-429X-15-68 23919374 PMC3750927

[B24] Niendorf T Hardy CJ Giaquinto RO Gross P Cline HE Zhu Y Kenwood G Cohen S Grant AK Joshi S Rofsky NM Sodickson DK Toward single breath-hold whole-heart coverage coronary MRA using highly accelerated parallel imaging with a 32-channel MR system Magn Reson Med 2006 56 167 76 10.1002/mrm.20923 16755538

[B25] Hays AG Hirsch GA Kelle S Gerstenblith G Weiss RG Stuber M Noninvasive visualization of coronary artery endothelial function in healthy subjects and in patients with coronary artery disease J Am Coll Cardiol 2010 56 1657 65 10.1016/j.jacc.2010.06.036 21050976

[B26] Terashima M Meyer CH Keeffe BG Putz EJ de la Pena-Almaguer E Yang PC Hu BS Nishimura DG McConnell MV Noninvasive assessment of coronary vasodilation using magnetic resonance angiography J Am Coll Cardiol 2005 45 104 10 10.1016/j.jacc.2004.09.057 15629383

[B27] Ehara S Nakamura Y Matsumoto K Hasegawa T Shimada K Takagi M Hanatani A Izumi Y Terashima M Yoshiyama M Effects of intravenous atrial natriuretic peptide and nitroglycerin on coronary vasodilation and flow velocity determined using 3 T magnetic resonance imaging in patients with nonischemic heart failure Heart Vessels 2013 28 596 605 10.1007/s00380-012-0292-z 23014927

[B28] Fischer SE Wickline SA Lorenz CH Novel real-time R-wave detection algorithm based on the vectorcardiogram for accurate gated magnetic resonance acquisitions Magn Reson Med 1999 42 361 70 10.1002/(SICI)1522-2594(199908)42:2<361::AID-MRM18>3.0.CO;2-9 10440961

[B29] Cunningham CH Pauly JM Nayak KS Saturated double-angle method for rapid B1+ mapping Magn Reson Med 2006 55 1326 33 10.1002/mrm.20896 16683260

[B30] Pruessmann KP Weiger M Scheidegger MB Boesiger P SENSE: Sensitivity encoding for fast MRI Magn Reson Med 1999 42 952 62 10.1002/(SICI)1522-2594(199911)42:5<952::AID-MRM16>3.0.CO;2-S 10542355

[B31] Stuber M Botnar RM Danias PG Sodickson DK Kissinger KV Van Cauteren M De Becker J Manning WJ Double-oblique free-breathing high resolution three-dimensional coronary magnetic resonance angiography J Am Coll Cardiol 1999 34 524 31 10.1016/S0735-1097(99)00223-5 10440168

[B32] Deimling M Heid O Magnetization prepared true FISP imaging Annual Meeting of the Society of Magnetic Resonance 1994 1994 San Francisco 495

[B33] Etienne A Botnar RM van Muiswinkel AMC Boesiger P Manning WJ Stuber M “Soap-Bubble” visualization and quantitative analysis of 3D coronary magnetic resonance angiograms Magn Reson Med 2002 48 658 66 10.1002/mrm.10253 12353283

[B34] Deyo RA Diehr P Patrick DL Reproducibility and responsiveness of health status measures statistics and strategies for evaluation Control Clin Trials 1991 12 S142 58 10.1016/S0197-2456(05)80019-4 1663851

[B35] Deshpande VS Cavagna F Maggioni F Schirf BE Omary RA Li D Comparison of gradient-echo and steady-state free precession for coronary artery magnetic resonance angiography using a gadolinium-based intravascular contrast agent Invest Radiol 2006 41 292 8 10.1097/01.rli.0000186566.38619.6d 16481912

[B36] Reeder SB Wintersperger BJ Dietrich O Lanz T Greiser A Reiser MF Glazer GM Schoenberg SO Practical approaches to the evaluation of signal-to-noise ratio performance with parallel imaging: Application with cardiac imaging and a 32-channel cardiac coil Magn Reson Med 2005 54 748 54 10.1002/mrm.20636 16088885

[B37] Greil GF Desai MY Fenchel M Miller S Pettigrew RI Sieverding L Stuber M Reproducibility of free-breathing cardiovascular magnetic resonance coronary angiography J Cardiovasc Magn Reson 2007 9 49 56 10.1080/10976640600897427 17178680

[B38] Cheng L Gao Y Guaricci AI Mulukutla S Sun W Sheng F Foo TK Prince MR Wang Y Breath-hold 3D steady-state free precession coronary MRA compared with conventional X-ray coronary angiography J Magn Reson Imaging 2006 23 669 73 10.1002/jmri.20567 16568438

[B39] Chang S Cham MD Hu S Wang Y 3-T navigator parallel-imaging coronary MR angiography: targeted-volume versus whole-heart acquisition Am J Roentgenol 2008 191 38 42 10.2214/AJR.07.2503 18562722 PMC3641883

[B40] van Elderen SGC Versluis MJ Westenberg JJM Agarwal H Smith NB Stuber M de Roos A Webb AG Right Coronary MR Angiography at 7 T: a direct quantitative and qualitative comparison with 3 T in young healthy volunteers1 Radiology 2010 257 254 9 10.1148/radiol.100615 20851943 PMC2941721

[B41] Zhu Y Hardy CJ Sodickson DK Giaquinto RO Dumoulin CL Kenwood G Niendorf T Lejay H McKenzie CA Ohliger MA Rofsky NM Highly parallel volumetric imaging with a 32-element RF coil array Magn Reson Med 2004 52 869 77 10.1002/mrm.20209 15389961 PMC2819016

